# Metabolite Profile of Alzheimer’s Disease in the Frontal Cortex as Analyzed by HRMAS ^1^H NMR

**DOI:** 10.3389/fnagi.2018.00424

**Published:** 2019-01-09

**Authors:** Yuzhong Zhang, Zhou Liu, Bing Ji, Lijian Liu, Shaoxiong Wu, Xiaowu Liu, Silun Wang, Liya Wang

**Affiliations:** ^1^Department of Radiology, The People’s Hospital of Longhua, Shenzhen, China; ^2^Graduate School, Medical College of Nanchang University, Nanchang, China; ^3^Department of Radiology and Imaging Sciences, Emory University School of Medicine, Atlanta, GA, United States; ^4^Department of Chemistry, NMR Research Center, Emory University, Atlanta, GA, United States; ^5^Yiwei Medical Technology, Inc., Shenzhen, China

**Keywords:** nuclear magnetic resonance, Alzheimer’s disease, metabolic change, neurochemistry, brain

## Abstract

**Background:** Investigation on neurochemical changes in the frontal cortex in individuals with Alzheimer’s disease (AD) and different Apolipoprotein E (APOE) genotypes, using *ex vivo* solid-state high-resolution NMR analysis, may lead to a better understanding of the neurochemistry associated with AD as well as new AD-specific metabolite biomarkers that might potentially improve the clinical diagnosis of AD.

**Methods:** Intact tissue samples of the frontal cortex were obtained from 11 patients and 11 age-matched non-demented controls. Metabolite profiles in all samples were analyzed *ex vivo*, using solid-state high-resolution magic angle spinning (HRMAS) 600 MHz ^1^H nuclear magnetic resonance (NMR). A logistic regression analysis was used to rank metabolites based on their level of contribution in differentiating the AD patient tissues and the controls, and different AD-associated APOE genotypes (APOE ε4 vs. APOE ε3).

**Results:** Tissue samples from the AD patients showed significantly lower NAA/Cr (*p* = 0.011), Ace/Cr (*p* = 0.027), GABA/Cr (*p* = 0.005), Asp/Cr (*p* < 0.0001), mI/Cr (*p* < 0.0001), and Tau/Cr (*p* = 0.021), and higher PCho/Cr (*p* < 0.0001), GPCho/Cr (*p* < 0.0001), and α&β-Glc/Cr (*p* < 0.0001) than the controls did. Specifically, a newly observed resonance at 3.71 ppm, referred to as α&β-Glc, was observed in 90.9% of the AD samples (10/11). Samples with APOE ε4 also exhibited higher PCho/Cr (*p* = 0.0002), GPCho/Cr (*p* = 0.0001), α&β-Glc/Cr (*p* < 0.0001), and lower Asp/Cr (*p* = 0.004) and GABA/Cr (*p* = 0.04) than the samples with APOE ε3 did. In the logistic regression analysis, PCho, GPCho, ASP, and α&β-Glc were found to be the most relevant metabolites for differentiating the AD patient tissues and the controls, and different APOE genotypes.

**Conclusion:** HRMAS ^1^H NMR with high spectral resolution and sensitivity offers a powerful tool to gain quantitative information on AD associated neurochemical changes. There are important neurochemical differences in the frontal cortex between the AD patient tissues and the controls, and between those with different APOE genotypes. The resonance (α&β-Glc) found at 3.71 ppm in the AD patient tissues may be further investigated for its potential in the diagnosis and monitoring of AD.

## Introduction

Alzheimer’s disease (AD) is one of the most prominent age-related disorders, characterized by progressive neuro-degeneration and has widespread social and economic implications (Alzheimer’s Association, 2012). It is thought that multiple biochemical, genetic and environmental factors contribute to AD. AD brains are characterized by hallmark neuropathologic changes, including neuronal loss, the formation of senile plaques containing the β-amyloid (Aβ) peptide and neurofibrillary tangles, which take place years prior to the onset of clinical manifestations (Braak and Braak, 1991; Scheltens et al., 2016).

At present, diagnosis of AD is challenging due to its insidious onset and primarily due to its reliance on clinical observations and cognitive examinations with neuropathologic confirmation post-mortem (Scheltens et al., 2016). Disease-specific neuroimaging biomarkers play increasingly central roles in the diagnosis of AD, prior to significant cognitive impairment, monitoring disease progression, and assessing responses to treatments (Dustin et al., 2016; Pini et al., 2016; Villemagne and Chetelat, 2016). Multiple studies have shown the potential of metabolic changes occurring in the temporal cortex and the hippocampus, as biomarkers for assisting diagnosis of AD (Block et al., 2002; Foy et al., 2011; Watanabe et al., 2012). However, only a few studies have reported that the cognitive decline induced by AD, is related to the neurochemical changes in the frontal lobe (Zhu et al., 2006; Siger et al., 2009; Graff-Radford et al., 2014). Additionally, the frontal lobe is an indispensable part of the functional network and performs many cognitive processes that often diminish along with the progression of AD (Finger et al., 2017). Imaging AD-specific neurochemical changes in the frontal cortex of preclinical AD brains, prior to significant cognitive impairment, may be useful to improve diagnosis and monitor disease progression.

Nuclear magnetic resonance spectroscopy (NMR or MRS) allows for the *in vivo* and *ex vivo* measurement of metabolites in the brain and can thus potentially enable the non-invasive quantification of neurochemical alterations to gain information on progressive neuro-degeneration in AD brains (Kuttner-Hirshler et al., 2017; Mullins et al., 2018; Yeh et al., 2018). In earlier studies, *in vivo* MRS was used to evaluate the level of a number of neurochemicals, such as *N*-acetyl aspartate (NAA), mI, Cr, and phosphocreatine (PC), Cho, and Cho derivatives, and Gln/Glu (Glx) in the AD patient tissues. This allows for the detection of subtle biochemical changes prior to clinical onset (Graff-Radford and Kantarci, 2013; Kantarci, 2013a,b). However, *in vivo* MRS studies are limited by low sensitivity and poor spectral resolution for the detection and differentiation of resonances of brain metabolites at a relatively low field and directly from patients, given the short acquisition time and intrinsic physiological motions encountered in clinical exams. Therefore, *in vivo* MRS is not sufficient to provide accurate metabolite quantification and detection of low concentration metabolites. To obtain a more comprehensive understanding of AD associated neurochemical changes, HRMAS ^1^H NMR spectroscopy methods operating at a ultrahigh field, i.e., >14T, has been applied to examine metabolite profiles in intact tissue specimens (Holmes et al., 2006; Dietz et al., 2017; Kaebisch et al., 2017). This powerful analytical method offers high sensitivity, exquisite spectral resolution, and a quantitative approach in detecting neurochemicals in the tissue, making it an attractive tool for investigating disease related metabolic changes and discovering metabolite biomarkers that can potentially be used for *in vivo* diagnosis.

In this study, we investigated the abnormalities of metabolite profiles in the frontal lobe to better understand the neurochemical association with AD in *ex vivo* HRMAS NMR. We also examined new metabolite profiles to identify AD-specific metabolite biomarkers that might potentially improve the clinical diagnosis of AD and APOE genotypes.

## Materials and Methods

### Tissue Samples

Post-mortem tissue samples from the frontal cortex of AD (*n* = 11, 5 females and 6 males with an average age of 63.9 ± 8.3 years) and gender- and age-matched non-demented control subjects (*n* = 11, 7 females and 4 males with an average age of 62.55 ± 9.16 years), were provided by the Tissue Bank of Emory University Alzheimer’s Disease Research Center, with the approval of the Institutional review board (IRB). All Alzheimer’s disease (AD) subjects were at the end-stage of the disease clinically and met the Consortium to Establish a Registry for AD (Mirra et al., 1991) and (Ball et al., 1997) for the neuropathological diagnosis of AD recommended by the National Institute of Aging.

All controls did not have any history of neurological diseases or significant neuropathological findings based on their medical records available to this study. In particular, control brains did not have senile plaques and had only sparse to moderate neurofibrillary tangles in limbic regions. The post-mortem intervals were 7.6 ± 4.1 and 8.1 ± 5.3 h for the AD and control groups, respectively. Tissue samples were frozen at –80°C immediately upon collection and stored at this temperature. In addition, samples from both the AD (*n* = 11) and control (*n* = 11) groups were divided into two groups based on the Apolipoprotein E (APOE) genotype status determined by the genetic test of APOE status: cases with no ε4 allele (APOE ε3) (*n* = 12, including 4 AD and 8 controls) and cases with at least one ε4 allele (APOE ε4) (*n* = 10, including 7 AD and 3 controls). Detailed demographic information is summarized in Table 1.

**Table 1:** Demographic and clinical features of tissue donors^∗^.

Characteristics	AD	Controls	APOE ε3	APOE ε4
Number (female/male)	44 (20/24)	33 (21/12)	40 (23/17)	37 (18/19)
Age at death (years)	63.91 ± 8.29	62.55 ± 9.16	64.1 ± 9.4	62.2 ± 7.8
PMI (hours)	7.64 ± 4.14	8.09 ± 5.37	8.5 ± 5.9	7.1 ± 2.8
Race (White/African American)	11 (9/2)	11 (8/3)	12 (11/1)	10 (6/4)
Age at onset (average)	49∼74 (55)	–	49∼64 (56)	52∼67 (56)
Years of dementia (average)	3∼17 (9.3)	–	6∼11 (9)	3∼17 (9.4)

*^∗^Mean ± standard deviations are presented for the age at death, post-mortem interval (PMI), age at onset and duration of dementia in each group.*

### HRMAS NMR Sample Preparation

A 200-mg frozen tissue block was obtained from the gray matter of the frontal lobe of each brain. Four sample blocks were taken from each AD tissue and each block was approximately 2 × 2 × 2 mm^3^. Three sample blocks were taken from each control tissue with the same size of the AD block. Overall, a total of 44 AD and 33 control tissue samples were used in the HRMAS NMR analysis. The spectral profile data from multiple samples from the same tissue block were consistent. However, there were some variations in the metabolite levels. While the test and retest of the same sample in a 12-h experiment interval gave typically less than 1% standard deviation (using the integral of the −CH_3_ resonance of NAA as a reference), the sample-to-sample deviations from the same tissue block were in the range of 3 – 10% (mean value of 5%), indicating a slight inhomogeneous metabolite distribution within a given brain region.

Subsequently, all the tissue blocks were analyzed individually with NMR. Each NMR sample was weighted (ranging from 31 to 38 mg) and then thawed in 99% D_2_O saline before being loaded on the sample holder/rotor (4 mm ZrO_2_). Caution was taken during the process in order to minimize mechanical damage to the sample. A ZrO_2_ insert (50 μl) was then placed in the sample holder to stabilize the sample and to provide balance for the rotor. D_2_O solvent was added to obtain the frequency-lock signal. An aliquot of TSP (sodium), or TSP, was added to the samples as the external reference for chemical shift and metabolite quantification. The NMR samples were prepared rapidly on ice to avoid possible sample degradation.

### HRMAS NMR Data Acquisition

HRMAS NMR experiments were conducted using a Bruker AVANCE 600WB solid-state NMR spectrometer (Bruker Instruments, Inc., Fremont, CA, United States) with a dedicated 4 mm HRMAS probe. The NMR probe head was pre-cooled to 4°C before loading the sample into the instrument. All experiments were carried out at 4°C. The sample/probe temperature was maintained throughout the experiment at 4 ± 0.1°C using a variable temperature control unit. Sample spinning rates were maintained in the range of 2800 KHz (±2 Hz). This sample spin rate was tested to ensure that the spin side-bands did not affect the spectrum window of interest. Spectra were acquired with and without suppression of the water signal. The pre-saturation of water was achieved with a zqpr sequence before acquisition pulses. A rotor-synchronized Carr–Purcell–Meibom–Gill (CPMG) pulse sequence [90-(τ-180-τ)*_n_* acquisition] was used as a T_2_-filter to suppress broad signals from the macromolecules. The inter-pulse delay (τ = 2π/ω_r_; τ indicates the sample spinning rate in time units and ω_r_/2 represents the spinning rate in kHz) was synchronized with the rotor rotation. The value of *n* for each sample was adjusted to create a T_2_-filter of 2*n*τ = 50 ms. The 90° pulse length was calibrated and adjusted based on each sample. The number of transients was typically 256. A repetition time of 5.0 s and a spectral width of 10 kHz were typically used. This procedure was adapted from that used in other studies (Cheng et al., 1997; Swanson et al., 2003). The spectra were processed using software installed on the instrument. A line to broaden the apodization of ^1^Hz was applied to all free induction decays (FIDs) before Fourier transformation. For resonance assignment and confirmation, two-dimensional (2D) ^1^H J-coupled gradient correlated spectroscopy (COSY) was used (Nagayama et al., 1980). COSY spectra were collected with a 6000 Hz spectral width (SW) in both dimensions (D1 and D2) and a 1.5 s relaxation delay. Thirty-two transients were averaged for each of the 256 increments during t1, corresponding to a total acquisition time of ∼4 h. Two-dimensional spectral data were analyzed using zero filling to a 1 × 1k matrix and weighted with a shifted square sine bell function and 1 Hz exponential line broadening for both D1 and D2 dimensions, followed by a Fourier transformation.

### Metabolite Assignment and Quantification

After all the spectra were Fourier transformed and the baseline offset corrected, chemical shift values of the resonances of interest were determined using –CH_3_ protons of TSP as the external reference of 0 ppm at 4°C. Resonance assignments for the metabolites of interest were obtained based on the chemical shifts reported in the literature (Martinez-Bisbal et al., 2004), and some were further confirmed by 2D COSY spectra. The spectral region from 1.5 – 4.5 ppm was the region of interest (ROI) for analysis. Peak integrals were measured for the selected resonances. The absolute concentration of each metabolite of interest was then referenced to the integral of –CH_3_ protons of TSP and calculated according to the Equation 1:

(1) $$C_{\text{m}}=9\times \left(\frac{I_{\text{m}}}{N_{\text{m}}}\right)\times C_{\text{TSP}}/(I_{\text{TSP}}\times W)$$

Where C_m_ = metabolite concentration, I_m_ = integral of the selected peak from a metabolite, N_m_ = number of protons in the selected peak of the metabolite, C_TSP_ = concentration of the external reference TSP (unit in mM), I_TSP_ = integral of TSP peak at 0 ppm, factor of 9 is used since the TSP has nine protons, and W = weight of the sample (unit in mg). In order to compare the results of this study to those of previous *in vivo* MRS studies, in which the resonance of Cr was typically used as the internal reference, even though it is recognized that the absolute concentration of Cr may also change in AD brain, we also presented the data as a ratio of the metabolite to Cr. Integrals per proton for each resonance selected to represent each metabolite of interest, were normalized against the −CH_3_ of Cr (3.03 ppm). The concentration of NAA was calculated as the sum of NAA and Ace in order to estimate the original NAA value before death.

### Statistical Analysis

The metabolite concentration data were averaged over multiple samples of each tissue block, with the results expressed as the mean ± standard deviation (SD). The mean value of each metabolite for different groups was then obtained by averaging each metabolite level from each subject in the group. The statistical difference between the AD and control groups, or the APOE ε4 and APOE ε3 groups, were analyzed using an independent two-tailed *t*-test with the SPSS 15.0 statistical package (LEAD Technologies, Inc., United States), with a *p* ≤ 0.05 indicating a difference with a statistical significance. Descriptive statistics followed by a Crosstabs analysis was performed to analyze the sensitivity and specificity of selected metabolite markers. The magnitudes of metabolic differences between the AD patient tissues and the controls, or APOE ε4 in AD and APOEε3 in controls, were defined as (AD_metabolites_/Control_metabolites_)−1, or (AD ε4_metabolites_/Control ε3_metabolites_)−1. Positive values indicate higher metabolite levels, while negative values indicate decreased metabolites in the AD samples, compared with the control samples, or the APOE ε4 samples, compared with the APOE ε3 samples. As a proof-of-principle and a feasibility-test for using NMR measured metabolite levels as biomarkers for AD, we selected a subset of metabolites as potential biomarkers of AD. A logistic regression (LR) algorithm, fitting a logistic function to the entire data set and further measuring the relationship between the classification label (AD vs. controls, and APOE ε4 vs. ε3) and metabolites, was used to sort the metabolites according to their contributions, to discriminate the AD from normal metabolites and APOE ε4 from ε3. The formula used is given in equation 2:

(2) $$p_{i}=\frac{1}{1+e^{-\sum_{j=1}^{M}\beta_{j}\chi_{ij}}}$$

Where *p*_i_ corresponds to the probability at observation *i*, β_j_ refers to the *j*^th^ regression coefficient, and *x*_ij_ is the *j*^th^ variable observation *i*.

## Results

**FIGURE 1:**
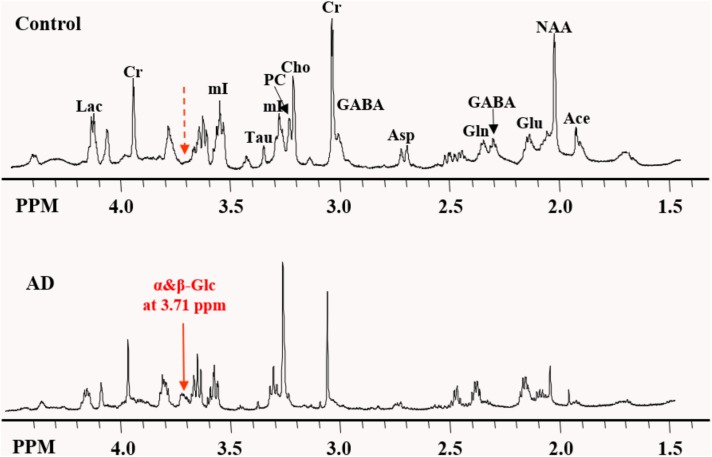
Expanded region of the HRMAS NMR spectra of tissue samples from the frontal cortex of the typical AD and control brains. The “signature” resonance of each metabolite is labeled. The newly observed AD-specific resonance (3.71 ppm) named as α&β-Glc in this study is indicated by the solid red arrow, while at the corresponding location of 3.71 ppm in control samples, no resonance peak was observed, as indicated by the dashed red arrow.

High-resolution NMR spectra with effective water suppression were obtained successfully from all samples. Figure 1 shows the expanded region (0.5 – 4.5 ppm) of the typical ^1^H HRMAS NMR spectra obtained from the frontal cortex of the AD and control group brains. The key resonances of major metabolites found in these brains were well resolved and identified and are consistent with those reported previously (Tsang et al., 2005). In addition, a new resonance arising at 3.71 ppm (noted as α&β-Glc) in the aromatic region, presumably from an aromatic metabolite, was found in 10 of 11 AD samples but in only 2 of 11 control samples, with a sensitivity of 91% and specificity of 82% in differentiating AD samples from the controls. In addition, specimens from the controls contained a considerably lower level of α&β-Glc than the AD specimens. This newly identified resonance, which has not previously been reported in other studies, appears to have a broadened line width. Two-dimensional COSY experiments confirmed no J-couple correlations between this signal and other resonances at the current sample concentration and resolution.

**FIGURE 2:**
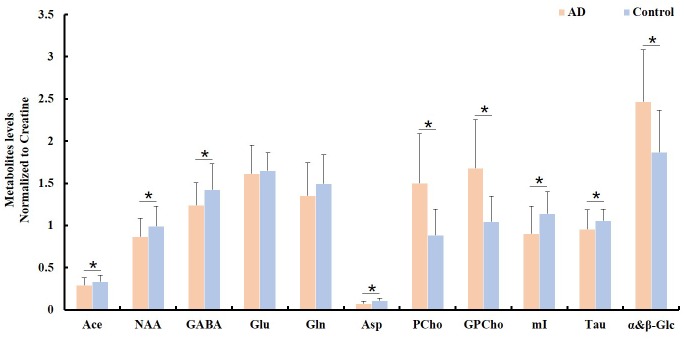
Comparison of different metabolite ratios (relative to creatine) calculated from the samples between the AD and control groups (^∗^indicating *p* < 0.05).

To examine the differences in the metabolite profiles between the AD and control groups, concentrations of selected metabolites from all samples within each tissue block were determined and averaged for each group. The ratios of the average metabolite levels normalized to Cr are shown in Figure 2. The AD samples showed significantly lower NAA/Cr (*p* = 0.011), Ace/Cr (*p* = 0.027), GABA/Cr (*p* = 0.005), Asp/Cr (*p* < 0.0001), mI/Cr (*p* < 0.0001), and Tau/Cr (*p* = 0.021) as well as higher PCho/Cr (*p* < 0.0001), GPCho/Cr (*p* < 0.0001), and α&β-Glc/Cr (*p* < 0.0001) than the control samples.

**FIGURE 3:**
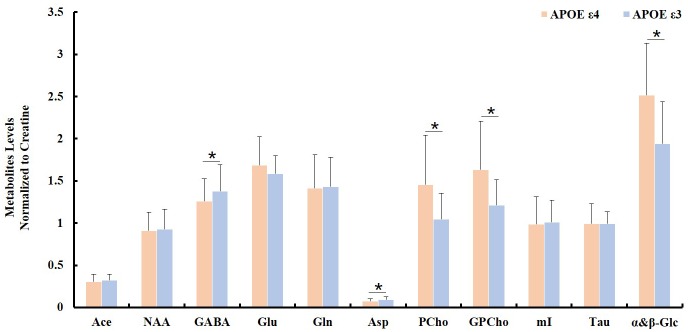
Comparison of ratios of metabolite/Cr between samples with no APOE allele (APOE ε3; *n* = 12) and with at least one APOE allele (APOE ε4; *n* = 10, ^∗^indicating *p* < 0.05).

**FIGURE 4:**
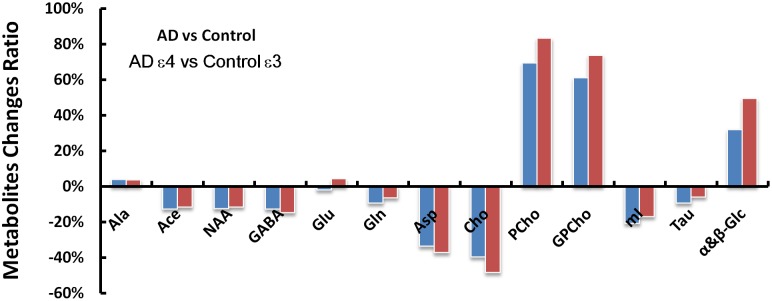
The AD and control groups were compared first without regard for the APOE genotypes (AD vs. Control) and then with only the APOE ε4 samples in the AD group and only the APOE ε3 samples in the control group (AD ε4 vs. Control ε3). Metabolic changes (AD_metabolites_/Control_metabolites_ - 1) found between the AD and control groups are greater in several metabolites (GABA, Asp, Cho, PCho, GPCho, and α&β-Glc at 3.71 ppm) when considering the APOE genotype of the samples with less pronounced differences in NAA, Gln, mI, and Tau.

Spectroscopic data were further analyzed to investigate the relationship between the APOE genotype and the metabolite levels in the samples of both the AD patient tissues and the controls. The results indicated that the APOE ε4 samples also exhibited higher PCho/Cr (*p* = 0.0002), GPCho/Cr (*p* = 0.0001), and α&β-Glc/Cr (*p* < 0.0001), and lower Asp/Cr (*p* = 0.004) and GABA/Cr (*p* = 0.04), than all APOE ε3 samples, as shown in Figure 3. When those samples with APOE ε4 allele in the AD group (*n* = 7) were compared to the APOE ε3 samples in the control group (*n* = 8), the magnitudes of the observed metabolic differences in PCho, GPCho, and α&β-Glc were even greater (as shown in Figure 4). The apparent AD-specific resonance (3.71 ppm) found in the aromatic region, α&β-Glc was exhibited in 8 of 10 APOE ε4 samples and 4 of 12 APOE ε3 samples with a sensitivity of 80% and specificity of 67% in differentiating APOE genotypes.

**FIGURE 5:**
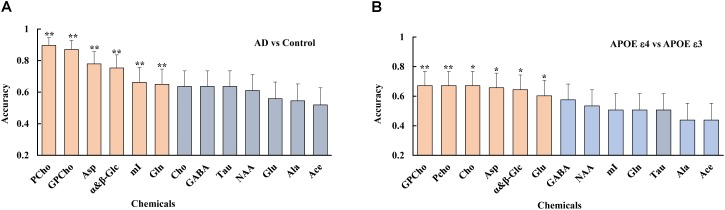
Using a logistic regression analysis, metabolites were ranked, such as PCho, GPCho, Asp, and α&β-Glc, were the top metabolites based on their contributions to both differentiate the AD subjects from the controls **(A)**, and classifying the different APOE genotypes **(B)**, with double asterisks and single asterisk indicating a *p* < 0.01 and *p* < 0.05, respectively.

A logistic regression analysis ranked the importance of each metabolite in differentiating the AD from the control group. The result showed that several metabolites, including PCho, GPCho, Asp, and α&β-Glc, were the metabolites significantly contributing to differentiating the AD subjects from the controls and classifying the APOE genotypes, as shown in Figure 5.

## Discussion

Currently, *ex vivo* NMR can mostly only be performed on tissue specimens obtained from post-mortem brains at the end stage of AD, but it can provide highly accurate and quantitative information compared to *in vivo* MRS, given the sensitivity an spectral resolution needed to study complex metabolism which involves many metabolites. Furthermore, it also provides an opportunity to identify subtle specific neurochemical changes in AD (Cheng et al., 2002; Choi et al., 2010), which may allow for the detection of new metabolite markers for an improved diagnosis and more specific monitoring of the progression of AD.

Instead of focusing on other cortical regions, such as the posterior cingulate cortex (Lim et al., 2012; Shiino et al., 2012; Wang et al., 2012; Watanabe et al., 2012; Fayed et al., 2014; Graff-Radford et al., 2014), parietal lobes (Azevedo et al., 2008; Siger et al., 2009), temporal lobe and the hippocampus (Block et al., 2002; Foy et al., 2011; Watanabe et al., 2012) as in previous studies, we focused on the frontal cortex in this study because of the crucial cognitive function impairment in the frontal cortex during AD (Finger et al., 2017).

Our data revealed significantly lower NAA+Ace/Cr (*p* = 0.017) and Asp/Cr (*p* = 0.001) in the AD patient tissues, which is consistent with previous *in vivo* MRS studies (Fayed et al., 2011, 2014; Lim et al., 2012; Wang et al., 2012). Solely produced in the mitochondria of neurons, NAA only exists within the cell bodies and axons of neurons (Wang et al., 2015) and can be regarded as a “marker” for neuronal density (Schuff et al., 2006). Since Asp acid is involved in the synthesis of NAA, our observation of lower Asp/Cr suggests that the decrease in NAA in AD may not only indicate the death of neurons but may also result from the perturbation of mitochondrial function. AD may cause a decrease in metabolites such as Asp acid in viable neurons, thus interrupting the synthesis of NAA and other neurochemicals. In line with the results of previous studies (Schuff et al., 2006; Wang et al., 2012; Graff-Radford et al., 2014), our results revealed an elevation in the level of Cho and its derivatives, i.e., PCho and GPCho, in the AD group, suggesting that cell membrane defects might be detected in AD, as PCho and Cho are involved in the turnover of the cell membranes (Ross et al., 1992). However, how Cho metabolism is interrupted in AD patients is not clearly understood and requires further investigation. In this regard, HRMAS NMR used in this study provides better-resolved spectra, which enables the differentiation of individual Cho derivatives and therefore offers an opportunity to further investigate AD-specific Cho metabolism in the future.

Results from the present study also showed that GABA/Cr, mI/Cr, and Tau/Cr were lower in the AD brains than in the controls. GABA is an inhibitory neurotransmitter critical to neuronal activities. Therefore, the reduction in GABA in the post-mortem AD samples most likely suggests an AD-related loss of capacity in this neurotransmitter system (Mann et al., 2009). mI plays the role of an osmolyte and has also been suggested to be a marker for glial content, as it has been found to be elevated in pathological states where glial activation is prominent (Bitsch et al., 1999). Previous *in vivo* MRS studies of the mI level in AD have been inconsistent with both reports of an increased (Burton et al., 2009; Graff-Radford et al., 2014) and decreased level (Wang et al., 2012; Su et al., 2016) in AD and mild cognitive impairment (MCI). In our study, mI was lower in the post-mortem AD brain samples. Whether the discrepancy in the current findings of mI is due to the use of post-mortem brain tissue or improved spectral resolution, that enables accurate quantification, is a subject which requires further investigation. We observed a reduction in the level of Tau, an β-amino acid, which is involved in osmoregulation as an osmolyte and a possible stabilizer of polar groups, on the surface of cellular membranes and proteins for cell volume adjustments. Tau is considered to be a protective agent in neurons exposed to many cell-damaging conditions, including oxidants, excitatory amino acids, hypoxia, and ischemic insults (Ripps and Shen, 2012). The reduced Tau level in AD in our study may indicate the loss of cellular functions induced by various pathological conditions.

APOE ε4 allele strongly predisposes humans to AD, with a 3.7-fold increase in the risk associated with the presence of one copy and a 12-fold increase in the risk associated with the presence of two copies, when compared with the risk in those with the ε3 isoform (Corder et al., 1993). The elevations in PCho/Cr and GPCho/Cr and the presence of the newly observed resonance (α&β-Glc) at 3.71 ppm, were more pronounced in the APOE ε4 samples than in the APOE ε3 samples. This possible correlation suggests the importance of examining the influence of underlying genetic factors on neurochemical changes in AD.

In order to assess the reproducibility of the data and the heterogeneity of the tissue samples, multiple samples from each 200-mg tissue block of each brain were analyzed and compared to determine possible differences between the subject groups. The ultra-high field strength (600MHz, ^1^H) used in this study provided spectra at high resolution with greater sensitivity, allowing for better resolution and quantification of metabolites. Such capability also enabled us to identify and assign additional resonances of metabolites that were not noticed in previous studies. Unlike a previous NMR AD study, which focused primarily on NAA and Cho compounds with a relatively small sample size (*n* = 7) (Cheng et al., 2002), we analyzed more specimens with more metabolites and discovered a new resonance peak of a metabolite (α&β-Glc) rising at 3.71 ppm specific to the AD specimens.

α&β-Glc was more frequently observed in the AD samples than in the controls (10/11 vs. 2/11), with a sensitivity of 91% and specificity of 82% in differentiating the AD from the controls, which merits further investigation for its potential as a diagnostic and prognostic marker of AD. However, the sensitivity and specificity dropped to 80% and 67%, when this newly observed resonance was used to predict the APOE genotypes (8/10 APOE ε4 samples vs. 4/12 APOE ε3 samples). Logistic regression showed that Asp, GPCho, PCho, and α&β-Glc are the top metabolites which contribute to differentiating both the AD sample from the healthy controls and the APOE ε4 from APOE ε3.

One of the limitations of the study was that relatively small numbers of the post-mortem AD samples and matched control samples were used, which limited the statistical power. Confirmation with more *in vivo* MRS studies in the future is needed. More cortical areas of the brain should be investigated using *ex vivo* NMR to generate a map of whole brain metabolic changes induced by progressive degeneration in AD patients. Additionally, validation with samples from patients with non-AD cognitive impairment will help to test the specificity of this metabolite combination.

## Conclusion

HRMAS ^1^H NMR, with high spectral resolution and sensitivity, offers a powerful tool for exploring neurochemical changes and identifying new metabolic biomarkers in AD. There are important neurochemical differences in the frontal cortex between the AD tissues and the controls, as well as between those different APOE genotypes. A new resonance (α&β-Glc) rising at 3.71 ppm needs to be further validated for its potential as a new metabolite marker of AD to improve the non-invasive diagnosis and monitoring of the progression of AD using MRS.

## Author Contributions

LW contributed to project idea and supervision, manuscript revision, integrity of this manuscript. YZ implemented the initial study and reviewed the manuscript. ZL implemented the study, analyzed the data, and drafted the manuscript. BJ analyzed the NMRS data. LL and SWu collected the NMR data. XL and SWa contributed to statistical analysis and reviewed the manuscript. All authors had reviewed this manuscript critically and approved its final submission.

## Conflict of Interest Statement

XL and SWa are employed by Yiwei Medical Technology, Inc. The remaining authors declare that the research was conducted in the absence of any commercial or financial relationships that could be construed as a potential conflict of interest.

## Abbreviations

Ace: acetate
Ala: alanine
APOE ε3: the 3 allele of Apolipoprotein E
APOE ε4: the 4 allele of Apolipoprotein E
Asp: aspartic
Cho: Choline
COSY: J-coupled correlation spectroscopy
Cr: Creatine
GABA: γ-aminobutyric acid
Gln: glutamine
Glu: glutamate
GPCho: glyocerphosphocholine
HRMAS: high resolution magic angle spinning
mI: myo-inositol
MRS: magnetic resonance spectroscopy
NAA: N-acetyl-aspartate
NMR: nuclear magnetic resonance
PCho: phosphocholine
Tau: Taurine
TSP: trimethylsilyl-2,2,3,3-tetradeuteropropionicacid
α&β-Glc: assigned resonance at 3.75 ppm
